# Surface Design of Antifouling Vascular Constructs Bearing Biofunctional Peptides for Tissue Regeneration Applications

**DOI:** 10.3390/ijms21186800

**Published:** 2020-09-16

**Authors:** Radoslava Sivkova, Johanka Táborská, Alain Reparaz, Andres de los Santos Pereira, Ilya Kotelnikov, Vladimir Proks, Jan Kučka, Jan Svoboda, Tomáš Riedel, Ognen Pop-Georgievski

**Affiliations:** Institute of Macromolecular Chemistry, Czech Academy of Sciences, Heyrovsky sq. 2, 162 06 Prague, Czech Republic; kucerova@imc.cas.cz (J.T.); alainreparaz@gmail.com (A.R.); santospereira@imc.cas.cz (A.d.l.S.P.); kotelnikov@imc.cas.cz (I.K.); proks@imc.cas.cz (V.P.); kucka@imc.cas.cz (J.K.); svoboda@imc.cas.cz (J.S.); riedel@imc.cas.cz (T.R.)

**Keywords:** biomimetic surface, hierarchical bioactive polymer brushes, vascular graft, “click”-chemistry, RGD peptide, X-ray photoelectron spectroscopy

## Abstract

Antifouling polymer layers containing extracellular matrix-derived peptide motifs offer promising new options for biomimetic surface engineering. In this contribution, we report the design of antifouling vascular grafts bearing biofunctional peptide motifs for tissue regeneration applications based on hierarchical polymer brushes. Hierarchical diblock poly(methyl ether oligo(ethylene glycol) methacrylate-*block*-glycidyl methacrylate) brushes bearing azide groups (poly(MeOEGMA-*block*-GMA-N_3_)) were grown by surface-initiated atom transfer radical polymerization (SI-ATRP) and functionalized with biomimetic RGD peptide sequences. Varying the conditions of copper-catalyzed alkyne-azide “click” reaction allowed for the immobilization of RGD peptides in a wide surface concentration range. The synthesized hierarchical polymer brushes bearing peptide motifs were characterized in detail using various surface sensitive physicochemical methods. The hierarchical brushes presenting the RGD sequences provided excellent cell adhesion properties and at the same time remained resistant to fouling from blood plasma. The synthesis of anti-fouling hierarchical brushes bearing 1.2 × 10^3^ nmol/cm^2^ RGD biomimetic sequences has been adapted for the surface modification of commercially available grafts of woven polyethylene terephthalate (PET) fibers. The fiber mesh was endowed with polymerization initiator groups via aminolysis and acylation reactions optimized for the material. The obtained bioactive antifouling vascular grafts promoted the specific adhesion and growth of endothelial cells, thus providing a potential avenue for endothelialization of artificial conduits.

## 1. Introduction

Cardiovascular disease is the most common cause of death worldwide. It is increasingly responsible for mortality related to stroke, heart failure, hypertensive emergencies, etc. [1] Deprivation of blood supply due to vascular dysfunction caused, e.g., by atherosclerosis leads to ischemia in the affected tissue and to organ failure, which can lead to severe life-threatening conditions, heart attack and stroke being the most serious. Bypass grafting is considered the gold standard for treating damaged vessels and arteries. The limited availability of healthy autologous vessels for bypass grafting procedures led to the fabrication of prosthetic vascular conduits. The overall conduit design is essential for the success of the implantation as the bulk material properties have to be tuned to mimic the features of the original tissue [2,3].

Grafts based on synthetic polymers, such as polytetrafluoroethylene (PTFE) and polyurethanes, are used [4], however their application in the bypass of small-diameter vessels (below about 5 mm, e.g., coronary arteries) is severely hindered by their poor blood compatibility [5]. Blood contact with practically any synthetic material leads to non-specific protein adsorption, which triggers the activation of platelets and the coagulation cascade [6]. This results in thrombus formation and leads to occlusion of the lumen of the graft [7]. Therefore, crucial step in development of effective small-diameter artificial vessels is to improve the hemocompatibility of their surface. In this regard, various surface modifications of artificial vessels have been applied to decrease thrombogenicity [8,9]. Heparin coatings have shown some success in improving graft patency [10,11], while currently, impregnation of the inner graft surface with pyrolytic carbon is often employed [12]. The latter approach promotes the stable binding of a passivating layer of protein, inhibiting the triggering of thrombogenicity [13].

However, until now only an intact layer of vascular endothelium can be considered as fully non-thrombogenic. For this reason, the development of vascular tissue engineering strategies has gained attention, harnessing the cells/humans’ own regeneration capacity. In particular, in situ regeneration strategies are based on inducing the adhesion and proliferation of endothelial cells to generate a lining of healthy non-thrombogenic endothelium on the synthetic vessel graft surface (i.e., endothelialization) [14,15].

In order for the graft to support in situ endothelialization, the surface must: (i) prevent non-specific protein adsorption (protein fouling), (ii) suppress material-mediated thrombosis and inflammation, and (iii) deliver bioactive signals guiding the cell–conduit interactions in manner similar to the extra-cellular matrix (ECM) [16]. Protein fouling on the graft surface upon implantation plays a central role in triggering subsequent deleterious responses, as well as blocking any specific cell-adhesion cues that might be added to the implant [6]. Therefore, the graft must be modified with a suitable antifouling, yet also functionalizable coating that will achieve the above stated requirements simultaneously [17].

The suppression of non-specific protein adsorption has been addressed through variety of coating strategies, including pre-adsorption of passivating protein layer, self-assembled monolayers of small functional organic molecules, and grafting of polymer chains [18]. So far, the most effective antifouling coatings consist of ultra-thin polymer brushes [19]. In these films, hydrophilic polymer chains are grafted by one end to a substrate in close proximity to each other, forcing them to stretch away from the surface. Their superior resistance to fouling has been associated with the entropic barrier that proteins have to overcome to adsorb at the surfaces as well as with the brush association with water. This ability is largely determined by the morphological properties of the polymer brush, layer thickness, high grafting density, and chain configuration [20,21]. Such brushes are prepared by surface-initiated controlled polymerizations such as atom transfer radical polymerization (SI-ATRP). In particular, polymer brushes prepared from carboxybetaine acryl- and methacrylamide [22,23,24], *N*-(2-hydroxypropyl) methacrylamide [22,25,26], and oligo(ethylene glycol) methyl ether methacrylate (MeOEGMA) [27] grown by SI-ATRP have demonstrated a significant suppression of fouling from several biological fluids, including blood plasma. Importantly, their ability to suppress protein fouling has been associated with marked reductions in surface thrombogenicity, adhesion of blood cellular components (erythrocytes, leukocytes and platelets) and fibroblasts, as well as the prevention of bacterial surface adhesion and colonization [28,29]. However, surface-initiated polymerizations require attachment of initiator to the conduit surface which in many cases due to the chemical inertness of the material and/or the need of preserving the conduit’s integrity and mechanical stability can pose significant issues [30]. This can be overcome with the usage of an anchor layers such as polydopamine (PDA) which available amine and catechol groups can be used for initiator attachment and follow-up “grafting-from” polymerizations [31]. PDA is, however, showing very low chemocompatibility [32], limited long term stability and exerts very high surface roughness, which in time could lead to disturbing the continuity of the polymer layer.

In addition to prevention of non-specific protein adsorption and associated deleterious phenomena, coatings for endothelialization of vascular grafts should provide bioactive signals that promote the desired cell–material interactions. This can be achieved by the immobilization of short peptide sequences that mimic motifs present in ECM proteins. In this regard, polymer brushes offer a versatile platform due to the abundance of polymer side groups that can be targeted for chemical modification. Typically, post-polymerization reactions are performed on brushes to endow them with activated ester functional groups [33,34], which can be subsequently easily functionalized with amine-containing molecules [35]. Nevertheless, the choice of an appropriate biofunctionalization procedure is critical in order to maintain the antifouling properties of the initial brush, avoiding harsh reaction conditions and unwanted residual reactivity that would lead to subsequent side reactions. Reactions classified as “click” chemistry have been successfully applied for this purpose [36,37,38,39]. Moreover, controlling the extent and distribution of immobilized bioactive elements is possible by tuning the polymer architecture. Previously, we employed a hierarchical polymer brush architecture consisting of a bottom chemically inert antifouling block and a top block of tunable thickness capable of participating in biofunctionalization reactions, which helped to maintain the layer’s fouling resistance after the immobilization of bioactive elements [39].

The hierarchical poly(MeOEGMA-*block*-GMA) brush bearing azide functionality enabled the precise control of the extent of chemical modification on the top reactive block while preserving the chemical inertness and the antifouling properties of the parent poly(MeOEGMA) brush. Importantly, a similar brush employing the same architecture was used to immobilize an ECM-mimicking peptide to promote the specific adhesion of a model fibroblast cell line. It was shown that although the polymer brush coating itself prevented non-specific adhesion, biofunctionalization with the peptide sequence led to the formation of a confluent cell layer [40].

Nevertheless, growth of these polymer brushes “grafted-from” the surface via SI-ATRP requires that the substrate present immobilized moieties capable of acting as ATRP initiators. Moreover, the surface design of biofunctional antifouling vascular constructs for tissue regeneration applications requires the selection of suitable bioactive signals to promote the cell–surface interactions. Enhanced endothelial cell adhesion followed by rapid endothelialization has been achieved by immobilization of either cell adhesive proteins, growth factors or bioactive peptides [41,42,43]. RGD, a fibronectin-derived tripeptide sequence (Arg-Gly-Asp), is widely distributed within extracellular matrix proteins, such as laminin and fibronectin, and has been recognized as a principal binding ligand of integrins, present in the membrane of endothelial cells [44,45,46]. Furthermore, various other peptide sequences and other types of receptors may be used to promote the surface immobilization of endothelial cells, such as aptamers and antibodies. 

Herein, we report a strategy for the surface modification of currently used vascular grafts with the aim of promoting endothelialization without triggering non-specific protein adsorption and associated deleterious responses. In light of the materials currently used in the design of vascular grafts, we selected an aminolysis reaction to impart the required polymerization initiator groups on the surface of currently available PET vascular grafts with the aim of growing antifouling yet also functionalizable hierarchical polymer brushes. We studied the biofunctionalization of such coatings with RGD peptide first on model silicon, glass, and gold substrates in order to optimize the conditions, and rigorously characterized polymer brush composition, RGD surface concentration, antifouling properties, and the associated cell adhesion and proliferation. We then applied these conditions on woven PET grafts and further observed the response of endothelial cells on the surface-modified grafts. The combination of the antifouling properties of hierarchically structured polymer brush coatings and the biomimetic properties of ECM-derived peptide motifs led to the formation of a confluent and stable layer of endothelial cells on the graft. The developed strategy provides a tool for the design of new artificial constructs with enhanced hemocompatibility by the regeneration of a layer of endothelium.

## 2. Results and Discussion

The surface modification of PET vascular grafts for endothelialization was performed in a multi-step procedure. This procedure consisted of three main steps, namely surface immobilization of polymerization initiator, controlled polymerization of diblock polymer brush and incorporation of reactive azide groups, and finally, immobilization of cell-adhesive RGD (structure given in SI, Scheme S1) peptide via the copper-catalyzed alkyne-azide cycloaddition (CuAAC) “click” reaction. Before adopting this procedure on vascular grafts of complex morphology, the surface modification strategy was optimized on model substrates of silicon, glass, and gold to enable more straightforward and thorough characterization.

**Scheme 1:**
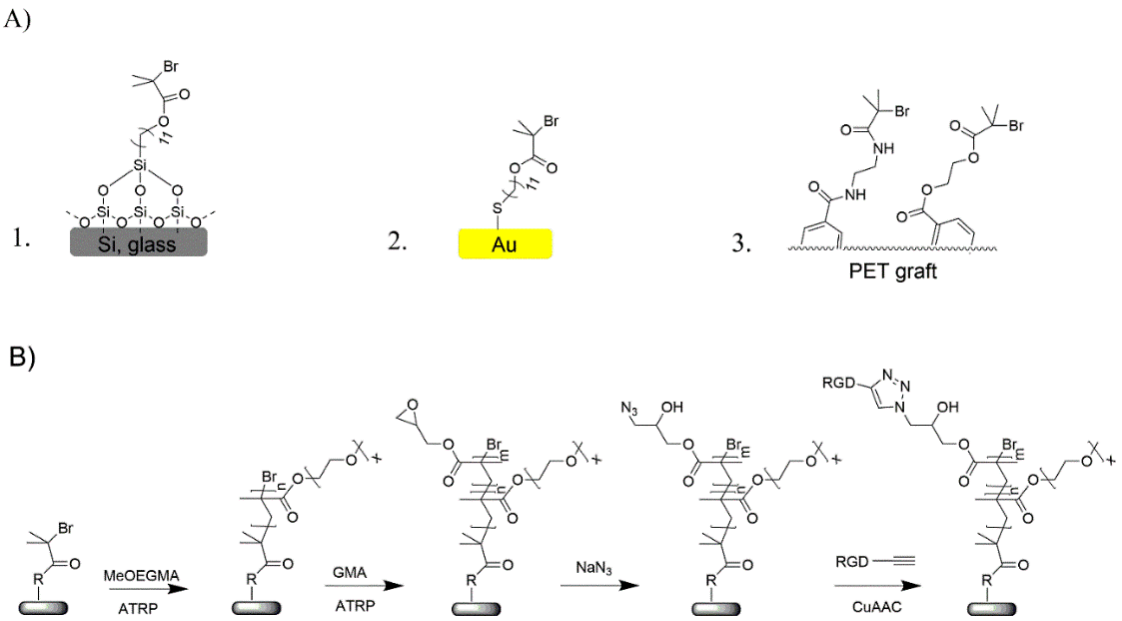
(**A**) detailed structure of the anchor layer R for the different substrates: 1. glass and silicon; 2. gold-coated chips for surface plasmon resonance (SPR); 3. PET graft; (**B**) Generalized route for the synthesis of bioactive hierarchical brush bearing RGD peptides. The structure of the RGD sequence is presented in SI, Scheme S1.

The procedure employed for the surface modification of vascular grafts and model substrates was almost identical, the only difference being the initial attachment of ATRP initiator moieties. On silicon and glass surfaces, a self-assembled monolayer (SAM) of 11-(2-bromo-2-methyl)propionyloxy)undecyltrichlorosilane molecules was used, while gold substrates were modified with a SAM of ω-mercaptoundecyl bromoisobutyrate (Scheme 1A). On the PET grafts, firstly hydroxy and amino functional groups were created by aminolysis and reacted with 2-bromoisobutyryl bromide (SI, Scheme S2). The choice of a diblock copolymer brush and method of preparation were based on the successful applications of similar coatings for fouling prevention. The following subsection describes the characterization and optimization performed on the model substrates.

### 2.1. Chemical Composition of the Surface Modification

Chemical characterization of the different stages in the surface modification procedure was carried out using X-ray photoelectron spectroscopy (XPS) on the model substrates and on the vascular graft. The attachment of the initiator SAM on Si is accompanied by the appearance of characteristic Br signals in the XPS spectrum (Figure S1), while identical signals were observed also on the initiator-functionalized vascular grafts. Subsequently, the hierarchical polymer brushes were synthetized and endowed with RGD peptides according to the general chemical route presented in Scheme 1. The bottom polymer block of poly(oligo(ethylene glycol) methyl ether methacrylate) (poly(MeOEGMA)) was grown under controlled radical polymerization conditions, which preserve the Br polymer end groups and thus allow the subsequent polymerization of second block bearing modifiable groups. MeOEGMA monomer was chosen to form the bottom block, as it can be polymerized via SI-ATRP in a well-controlled manner and the resulting polymer shows good antifouling performance. Based on results presented in previous reports, a dry thickness of 20 nm was selected for the poly(MeOEGMA) block as sufficient to provide good resistance to fouling from blood plasma [39,47]. In order to add to the antifouling polymer brushes reactive groups able to undergo the “click” reaction, the poly(MeOEGMA) was extended with a poly(GMA) top block. The thickness of the poly(GMA) block was tuned to 4 nm by adjusting the polymerization time. Subsequently, the epoxide rings of poly(GMA) were opened by reaction with NaN_3_ in order to introduce the “click”-reactive moieties to the top-block of the hierarchical brush (poly(MeOEGMA-*block*-GMA-N_3_)).

**Figure 1:**
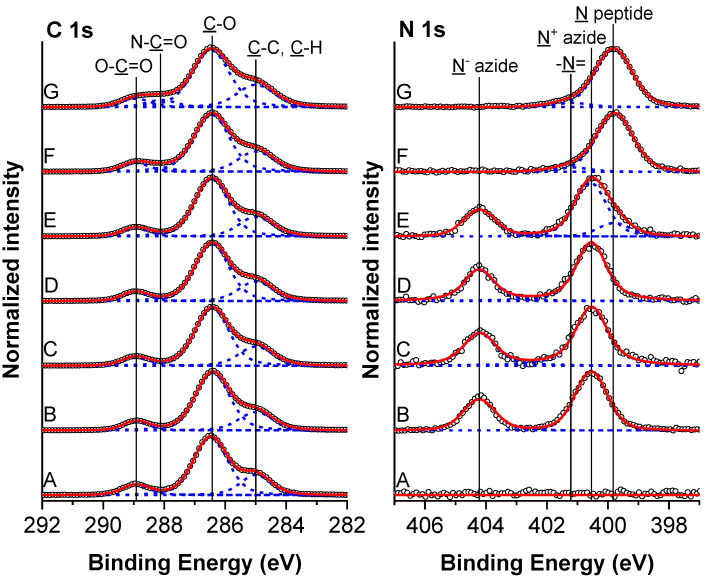
High resolution C 1s and N 1s XPS spectra of polymer brush poly(MeOEGMA-*block*-GMA) brush grown over Si substrates before (**A**) and after reaction with azide (**B**) and after immobilization of RGD peptide to surface concentration 1.1 nmol/cm^2^ (**C**), 12.1 nmol/cm^2^ (**D**), 3.2 × 10^2^ nmol/cm^2^ (**E**), 1.2 × 10^3^ nmol/cm^2^ (**F**) and 4.6 × 10^3^ nmol/cm^2^ (**G**). The composition of the layers is presented in Table S1.

**Figure 2:**
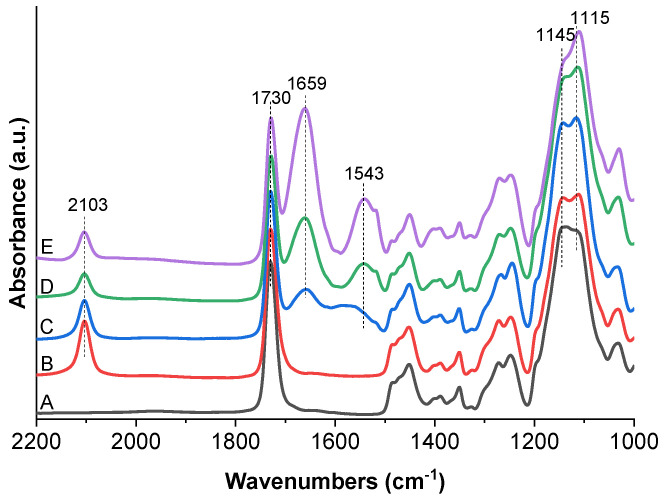
GAATR-FTIR spectra of polymer brush poly(MeOEGMA-*block*-GMA) brush grown over Si substrates before (**A**) and after reaction with azide (**B**) and after immobilization of RGD peptide to surface concentration 3.2 × 10^2^ nmol/cm^2^ (**C**), 1.2 × 10^3^ nmol/cm^2^ (**D**) and 4.6 × 10^3^ nmol/cm^2^ (**E**).

The composition of the prepared hierarchical polymer brushes was confirmed by XPS and grazing angle attenuated total reflection Fourier-transform infrared (FTIR-GAATR) spectroscopies. High resolution C 1s and N 1s spectra obtained on modified silicon wafers are presented in Figure 1. The composition of particular chemical states is reported in Table S1. The C 1s spectrum of hierarchical poly(MeOEGMA-*block*-GMA) polymer brush exhibited three characteristic peaks which are assigned to C–C (285.0 eV), C–O (286.5 eV) and O–C=O (288.9 eV) carbon species. The N 1s spectrum lacked any nitrogen contributions. The successful incorporation of azide groups (existing in the mesomeric –N=N^+^=N^−^ ⇆ –N^−^–N^+^≡N forms) in the poly(MeOEGMA-*block*-GMA-N_3_) brush was verified by the appearance of two peaks in N 1s spectra arising from the negative and positive nitrogens at 400.6 eV and 404.2 eV, respectively. The observed ratio of these two contributions (2.1:1.1) corresponded quite well to the expected theoretical ratio of 2:1. The azidation did not lead to any observable change in C 1s spectra, indicating a preserved structure of the poly(MeOEGMA) block. Successful attainment of the poly(MeOEGMA-*block*-GMA-N_3_) and its azidation were further corroborated by GAATR-FTIR. The IR spectrum of the poly(MeOEGMA-*block*-GMA) brush (Figure 2) showed the characteristic contributions of the methacrylate ester carbonyl group as a single intensive band at 1730 cm^−1^. In addition of the various CH_2_ modes in the fingerprint region, the spectra of the hierarchical brushes showed the C–O–C stretching vibrations at 1145 and 1115 cm^−1^ characteristic of the methacrylate fragments and the bottom block oligo(ethylene glycol) side chains. The ring opening of GMA units of the hierarchical poly(MeOEGMA-*block*-GMA) brushes and the incorporation of “clickable” functional groups by NaN_3_ led to the appearance of a distinctive asymmetric azide stretching band at 2103 cm^−1^ attributed to the incorporated functional groups.

**Figure 3:**
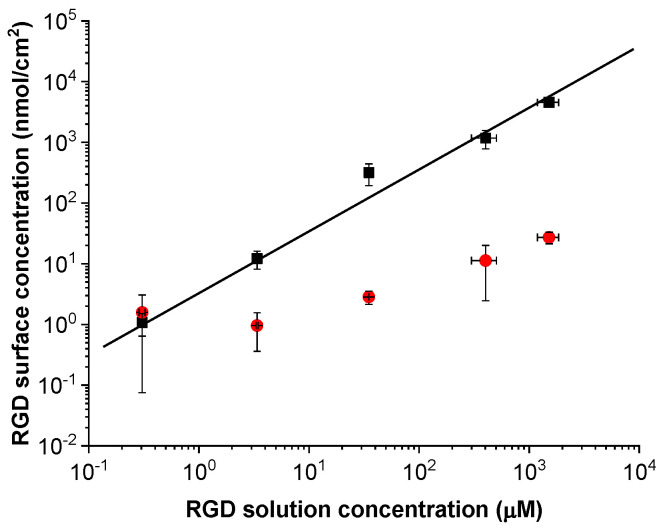
The surface concentration of RGD peptides (■) immobilized on poly(MeOEGMA-*block*-GMA-N_3_) brushes (grown over Si substrates) linearly increases with increasing the peptide’s solution concentration. The surface concentration of physisorbed RGD peptides (●) was probed utilizing the same reaction conditions, but in absence of Cu(II). The surface concentration of RGD peptides was determined directly using radio-assay and ^125^I-radiolabeled RGD-peptide.

The azide-containing polymer brushes were functionalized by copper-catalyzed “click”-reaction with the alkyne terminated RGD peptide. The “click”-approach is good choice for functionalization as it proceeds rapidly, even at low peptide concentrations, at ambient temperature in water without any side products, thus not introducing unwanted chemical changes in the original brush [38]. The reaction time for all experiments was kept at 15 min in order to suppress the non-covalent peptide bonding. In order to prepare samples with defined peptide concentrations for cell culture experiments, the dependence of the amount of immobilized RGD on the concentration of peptides in the reaction solution was studied using ^125^I-radiolabeled peptide derivative. The immobilized peptide concentrations were determined for solution concentrations ranging from 0.3 to 1.5 × 10^3^ μM. These experiments showed a direct dependence of the amount of covalently bound peptide on the peptide concentration in solution. The total amount of the surface immobilized peptides is linearly dependent on the solution concentration, as seen in the results from the radio-assay experiments (Figure 3). Parallel blank experiments were performed in which the reaction feed without the Cu(II) salt was left in contact with the surface for identical time to determine the amount of non-specifically bound peptides. The non-specific peptide binding is below 10% of the total amount of immobilized peptides for the RGD solution concentration of 3 μM, while it amounts to only 1% when the RGD peptide concentration is 30 μM or higher. This clearly shows the effectiveness of the “click”-chemistry ligation of RGD-peptide to the hierarchical polymer brushes. The samples used for cell studies, additional physico-chemical characterization, and the modification of the PET vascular graft were prepared with label-free peptides.

Further confirmation of the binding was obtained through chemical characterization of the surface modifications after biofunctionalization with RGD peptide. XPS and FTIR spectra of the biofunctionalized surfaces revealed the appearance and gradual increase in intensity of spectral features corresponding to the peptide incorporation. Moreover, no other changes to the polymer brush structure were observed, which is important for the preservation of its fouling resistance. The immobilization of RGD peptides on the poly(MeOEGMA-*block*-GMA-N_3_) hierarchical brushes should lead to a decrease of the azide signals and a concomitant rise of a new peak in N 1s spectra at 399.8 eV originating from amide NH–C=O nitrogen. At very low RGD surface concentrations, i.e., below 12.1 nmol/cm^2^ the expected amide contributions are not discerned, probably being buried under the tailing region of the N^−^ azide moieties at about 400.6 eV. Prominent changes in the N 1s spectra and identification off the RGD presence could be observed when the peptide surface concentrations rose above 3.2 × 10^2^ nmol/cm^2^. The azide contributions in the surface region dropped below the detection limits of the XPS measurement when the RGD surface concentration has risen above 1.2 × 10^3^ nmol/cm^2^. At high peptide surface concentrations, we could even identify a weak contribution at about 401.2 eV stemming from the presence of the –N= moiety of the triazole rings and the charged guanidinium groups. The immobilization of peptides was also accompanied with changes in the high-resolution C 1s spectra. Mainly the increase of RGD surface concentration led to decrease in the intensity of C–O peak and the rise of a well-defined new peak of amide group (NH–C=O, 288.1 eV). The same trends were observed in the XPS spectra of the polymer brush coatings synthesized on the substrates of gold (intended for protein fouling tests) and glass (for cell seeding experiments). Furthermore, no copper Cu residues which could lead to toxicity of the hierarchical brushes bearing RGD motifs could be detected (below the detection limit of XPS measurement of 0.1 atomic %).

FTIR spectroscopy measurements of the poly(MeOEGMA-*block*-GMA-N_3_) brush coating after the subsequent “click” reaction further confirmed the chemical changes associated with covalent peptide binding. Namely, the immobilization led to: (1) a decrease in intensity of the originally observed azide band; (2) the appearance of the characteristic Amide I and Amide II bands from the attached peptide at 1659 cm^−1^ and 1543 cm^−1^, respectively. The successful immobilization of peptides on the azide bearing hierarchical brush was observed via GAATR-FTIR down to surface concentrations of 3.2 × 10^2^ nmol/cm^2^. At the lower RGD surface concentrations (of only 12.1 and 1.1 nmol/cm^2^) the peptide presence did not lead to intense bands in the GAATR-FTIR spectra that could be reliably assigned. Taken together, the radio-assay, XPS and GAATR-FTIR findings prove the successful immobilization of the peptides on the hierarchical poly(MeOEGMA-*block*-GMA-N_3_) brushes.

### 2.2. Proving The Antifouling Properties of the Hierarchical Brushes

The surface of vascular grafts intended for tissue engineering and regeneration applications needs to support the adherence, growth, and proliferation of endothelial cells, while at the same time operating in contact with blood plasma. Hence, it must provide the present bioactive RGD motifs to adherent cells while preventing the nonspecific adsorption of blood proteins, which would elicit thrombosis and inflammation.

**Figure 4:**
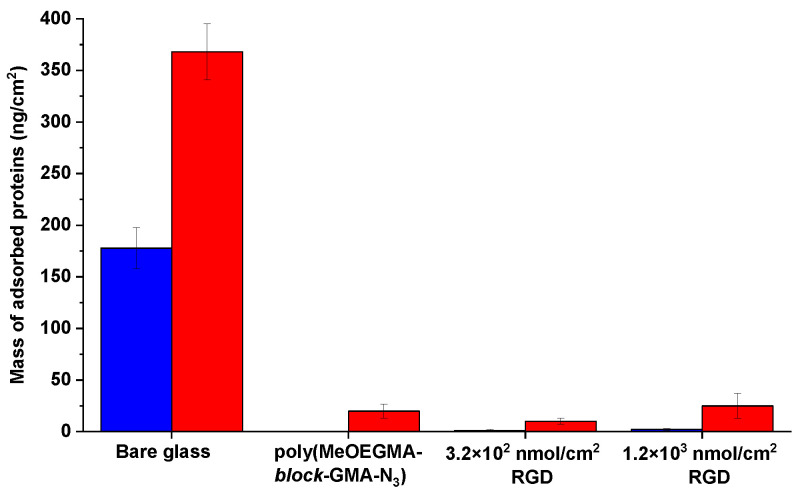
Comparison of the fouling from cell seeding medium (blue) and undiluted human blood plasma (HPB) (red): bare gold, poly(MeOEGMA-*block*-GMA-N_3_) polymer brush before and after immobilization of RGD peptide of surface concentration 3.2 × 10^2^ nmol/cm^2^ and 1.2 × 10^3^ nmol/cm^2^.

SPR measurements of fouling from cell-seeding medium and blood plasma were performed on poly(MeOEGMA-*block*-GMA-N_3_) coatings prepared on gold coated substrates. When challenged against the non-specific adsorption from cell seeding medium, both the pristine and RGD modified poly(MeOEGMA-*block*-GMA-N_3_) brushes reduced the adsorption to less than 3 ng/cm^2^. This is more than a 99% reduction in comparison to deposits on bare gold, thus proving the excellent antifouling properties of the hierarchical polymer brushes even after biofunctionalization (Figure 4). We further tested the RGD modified polymer brushes against fouling from blood plasma, as this represents the protein composition of the medium in which the vascular graft is meant to operate. The pristine nonfunctionalized poly(MeOEGMA-*block*-GMA-N_3_) brush reduced the fouling by 95% with respect to the uncoated substrate. The fouling resistance remained practically unchanged upon immobilization of the RGD sequence and did not surpass 25 ng/cm^2^ even when the peptide surface concentration was risen to 1.2 × 10^3^ nmol/cm^2^. This excellent fouling supports the effectiveness of combining an inert antifouling bottom block of poly(MeOEGMA) with a thin reactive poly(GMA-N_3_) segment in a diblock architecture. Despite the fact that the RGD sequences shift the surface charge of neutral surfaces to negative [48], this does not cause a significant deterioration of the initially observed protein repellence. Previously, polymer brushes displaying comparable antifouling properties were also shown to be highly hemocompatible and cause low surface trombogenicity. This suggests the potential of similar biofunctional coatings to be used for the design of vascular conduits, but further specific studies on the thrombogenicity in contact with whole blood are needed [29].

### 2.3. Proving the Biofunctionality of the Hierarchical Brushes

**Figure 5:**
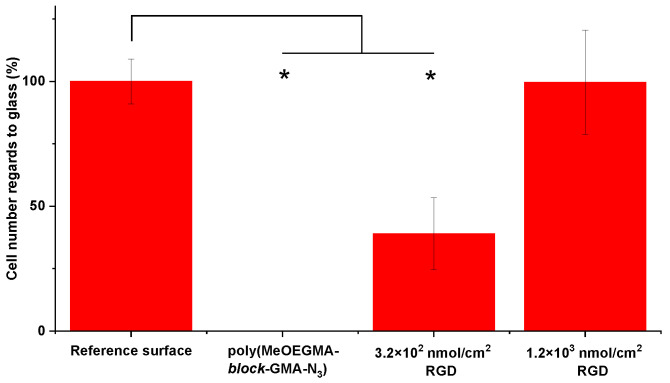
Relative number of HUVEC cells on tested surfaces determined from live/death assay. While the pristine poly(MeOEGMA-*block*-GMA-N_3_) brushes completely canceled out the cell adhesion and viability, the brush with 1.2 × 10^3^ nmol/cm^2^ RGD showed similar number of adherent and viable cells as the reference glass surface. Significant differences between the number of cells were calculated by the student t-test (* *p* < 0.05).

To assess the effectiveness of the peptide-functionalized poly(MeOEGMA-*block*-GMA-N_3_) hierarchical brushes to elicit specific endothelial cell adhesion we performed cell seeding tests utilizing HUVEC cells and counting adhering cells by live/dead assay. Cell seeding was performed for up to 3 days, with bare glass and non-functionalized poly(MeOEGMA-*block*-GMA-N_3_) brushes used as controls. Biofunctionalization of the polymer brushes with RGD peptide triggered the adhesion of the HUVEC cells (Figure 5). The number of cells on the surface was directly dependent on the RGD surface concentration. An RGD concentration on the brush surface of 1.2 × 10^3^ nmol/cm^2^ led to high adherence of viable HUVEC cells, on the same level with the glass surface. As the glass suffers from the rapid non-specific deposition of proteins present in the medium, it serves as a positive control of high cell adhesion. Interestingly, the hierarchical brushes with a lower RGD concentration of 3.2 × 10^2^ nmol/cm^2^ had a significantly lower number of surface-adherent cells than those with a higher RGD surface concentration (approx. 40%). Furthermore, pristine poly(MeOEGMA-*block*-GMA-N_3_) surfaces bearing no peptide did not induce any cell adhesion, even after three days of seeding in serum enriched medium. This highlights not only the excellent fouling resistance of the brush, but that varying the surface concentration of biomimetic moieties allows control over the cell-adhesive properties of surfaces.

**Figure 6:**
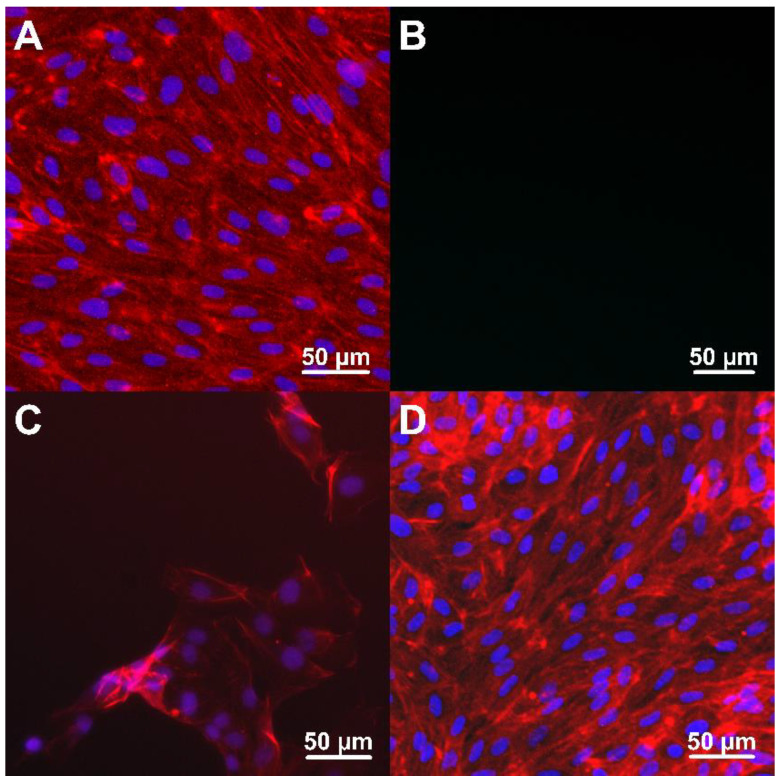
Adhesion of HUVEC cells on: reference bare glass sample (**A**), pristine poly(MeOEGMA-*block*-GMA-N_3_) brushes (**B**), brushes with 3.2 × 10^2^ nmol/cm^2^ (**C**) and 1.2 × 10^3^ nmol/cm^2^ (**D**) RGD peptides. The actin filaments (red) and cell nuclei (blue) were stained by Phalloidin–Atto 594 and Hoechst 33342, respectively.

To further analyze the nature of the cell adhesion on these surfaces we examined the morphology of HUVEC cells using confocal microscopy after fluorescence labeling (Figure 6). The obtained images confirmed the spreading of HUVEC adherent cells on the biomimetic surface at both concentrations of RGD. However, the staining process induced partial cell detachment from the hierarchical brush bearing of the lower (3.2 × 10^2^ nmol/cm^2^) RGD concentration, observed as a gradual washing out of the adherent HUVEC cells. Evidently, the lower RGD surface concentration was not sufficient to provide strong cell-surface interactions for stable cell attachment and cell growth over the antifouling polymer surface. In contrast, after 3 days of incubation the HUVEC cells on poly(MeOEGMA-*block*-GMA-N_3_) brushes bearing 1.2 × 10^3^ nmol/cm^2^ RGD formed a homogeneous, dense, and continuous layer. This proves that the presence of RGD fragments at the higher surface concentration also stabilized the HUVEC adhesion on the biofunctional hierarchical brush, promoting the growth and colonization of the artificial material bearing bioactive motifs.

The cell seeding tests on model glass substrates showed that the cell-adhesive character of the surface can be controlled by tuning the concentration of peptide on the brush surface. The strong response of the adherent HUVEC cells originates from the ability of the hierarchical brushes to suppress the nonspecific interactions and at the same time effectively display peptide sequences at the interface, without one of the behaviors negatively impacting the other. The ability to precisely control the surface concentration of RGD bioactive motifs resulted in control of HUVEC adhesion, stability, and colonization of the artificial material.

### 2.4. Applying the Biofunctional Antifouling Hierarchical Brush Coating Strategy to PET Vascular Grafts

**Figure 7:**
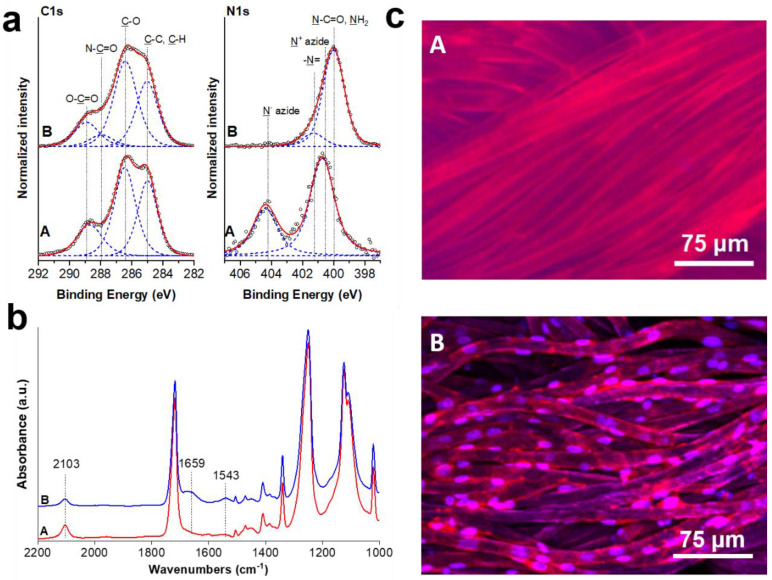
XPS (**a**) and GAATR-FTIR (**b**) characterization of PET conduits modified with antifouling hierarchical poly(MeOEGMA-*block*-GMA-N_3_) polymer brushes (**A**) and their biofunctional counterparts with 1.2 × 10^3^ nmol/cm^2^ RGD peptide (**B**). HUVEC cell adhesion (**c**) on the respectively modified PET conduits. Actin filaments (red) and cell nuclei (blue) were stained by Phalloidin–Atto 594 and Hoechst 33342, respectively.

Having characterized the structure of the hierarchical polymer brushes and proved their bioactivity in obtaining confluent and stable endothelial cell layers on the planar surfaces, we optimized the approach to modify the surface of woven PET vascular grafts (Scheme S2) and thus establish the necessary assets for the formation of healthy non-thrombogenic endothelium. Porosity and specific surface area of the vascular grafts were determined via mercury porosimetry and BET surface area analysis, respectively. The specific surface of the grafts was used to calculate the amount of peptide solution necessary to ensure identical reaction conditions as in the ones used on the model planar surfaces while maintaining the same concentration (see Experimental for further details). We chose the conditions used for obtaining biofunctional hierarchical poly(MeOEGMA-*block*-GMA-N_3_) polymer brush bearing 1.2 × 10^3^ nmol/cm^2^ RGD over the planar surface and applied them on the microporous PET graft. The successful synthesis was verified via XPS and GAATR-FTIR (Figure 7; for the detailed analysis of individual modification steps kindly refer to Figures S2–S5 and Table S2). Shortly, the aminolysis reaction resulted in anchoring amine and hydroxyl groups on the otherwise chemically inert woven vascular grafts. SEM analysis verified the uniform topography without disruptions of the fiber continuous structure (Figure S4). The presence of amine and amide groups on the surface of the PET led to the appearance of clear N 1s signals at about 400 eV in the high resolution XPS spectra. The incorporated reactive amine and resulting hydroxyl groups from the PET aminolysis were acylated with bromoisobutyrate bromide to provide SI-ATRP initiating moieties over the graft surface, corroborated in the Br 3d region of the XPS spectrum (Figure S2). In this way the hierarchical polymer brush could be grown directly from the graft surface via SI-ATRP polymerization without the need of an anchoring layer. The synthesis of hierarchical poly(MeOEGMA-*block*-GMA-N_3_) brushes led to significant changes of the originally measured C 1s spectra on the PET grafts, mainly through the appearance of the dominating C-O contributions at 286.5 eV and the characteristic N 1s features of the azide groups as in the case of the planar silicon surfaces. The subsequent immobilization of RGD peptides was proven by the appearance of amide peaks at 400 eV in the high-resolution N 1s spectrum and a concomitant broadening of the initial C(=O)-O peaks as a result of the presence of N-C=O contributions in the C 1s spectrum (Figure 7a). Notably the ratio between N-C(=O) peak in N 1s and C(=O)-O peak in C 1s region of the biofunctional hierarchical brush was determined to be 0.68 (Table S2). This value corresponds well to the value of 0.74 determined on planar silicon samples modified with biofunctional hierarchical poly(MeOEGMA-*block*-GMA-N_3_) polymer brushes bearing 1.2 × 10^3^ nmol/cm^2^ RGD (Supplementary Table S1), thus verifying the consistent amount of surface immobilized bioactive peptides. The incorporation of the biofunctional RGD peptides on the antifouling hierarchical brush grown from the activated PET woven conduit was further proven by GAATR-FTIR (Figure 7b). The “click” of the peptide led to drop in intensity of the azide contribution at about 2100 cm^−1^ and the appearance of the amide I and II bands at 1659 and 1543 cm^−1^, respectively. All surface modifications were restricted to the outermost surface of the individual fibers, thus preserving the initial integrity and mechanical stability of the woven PET material (Figure S3). Thus, the modification procedure developed on the planar substrates was adopted on the surface of PET woven vascular graft, providing essentially the same surface concentration of bound bioactive RGD moieties.

Finally, we tested the biofunctionality of the PET vascular conduit bearing RGD peptides by performing additional cell seeding experiments in identical conditions as the ones on planar glass substrates. The non-specific adhesion of HUVEC cells was completely suppressed on the PET conduit bearing the antifouling hierarchical poly(MeOEGMA-*block*-GMA-N_3_) polymer brushes (Figure 7c). The presence of 1.2 × 10^3^ nmol/cm^2^ RGD peptide sequences on these surfaces led to pronounced specific cell adhesion similar to the case of the planar samples. The displayed RGD sequences by the poly(MeOEGMA-*block*-GMA-N_3_) hierarchical brushes supported the adhesion and growth of the HUVEC cells. These cells appear to be spreading longitudinally along the individual modified fibers and even bridging between fibers that are in close proximity (Figure 7c and Figure S6). At the same time, cells adhered strongly to the woven conduits bearing RGD, remaining stably attached during the staining process. The HUVEC cells freely penetrated the woven mash and colonized the peptide-bearing PET conduit. Notably, both the lumen and the outer side of the artificial PET woven vessel bearing the biofunctional hierarchical poly(MeOEGMA-*block*-GMA-N_3_) polymer brushes bearing 1.2 × 10^3^ nmol/cm^2^ RGD have been colonized (Figure S6). The ability of adhering cells to closely follow the morphology of the modified substrates serves as a proof of concept for the envisioned endothelialization of the woven PET conduit. Furthermore, optimization in the design of grafts employing this type of coating may lead to better support for confluent cell layer. This is essential for in-situ regeneration and tissue engineering of a functional conduit.

The promotion of cell adhesion results from the immobilization of bioactive peptide RGD sequences while at the same time preserving the antifouling properties of the non-cytotoxic support. The perspective variation of the amount and type of bioactive peptides can further widen the possibilities of control over the biomimetic performance of vascular conduits for various biomedical applications. Although demonstrated only on PET woven fibers, the presented surface modification approach can be envisioned as a promising route for the modification of other polyester materials being used in medical implants.

## 3. Materials and Methods

### 3.1. Materials

CuBr_2_ (99.999% trace metal basis), CuBr (99.99%), 2,2’-bipyridyl (99%), oligo(ethylene glycol) methyl ether methacrylate (*M*_n_ = 300 g/mol, MeOEGMA), glycidyl methacrylate (97%, GMA), ethylenediamine (99.5%, EtDA), α-bromoisobutyryl bromide (98%, BiBB), copper sulphate (CuSO_4_), sodium L-ascorbate (98%), NaN_3_ (99.5%) were purchased from Sigma-Aldrich (Czech Republic). Deionized (DI) water was obtained from a Milli-Q purification system (Milli-Q gradient A10, Merck-Millipore). Tetrahydrofuran (THF, Lach-Ner, Czech Republic) was distilled under argon over sodium immediately prior use. All other organic solvents (Lach-Ner, Czech Republic) of analytical grade were used as received. ATRP initiators 11-(2-bromo-2-methyl)propionyloxy)undecyltrichlorosilane and ω-mercaptoundecyl bromoisobutyrate were synthesized according to procedures reported earlier [49,50]. Human blood plasma, Endothelial Cell Growth Medium 2 Kit, and HUVEC cells were obtained from Promocell (Germany). Penicillin-streptomycin (10 U/mL, 10 μg/mL), trypsin-EDTA (0.05%–0.02%) and Phalloidin–Atto 594 were from Sigma-Aldrich (Czech Republic). LIVE/DEAD™ Viability/Cytotoxicity Kit for mammalian cells and Hoechst 33342 were purchased from Thermo Fisher Scientific (USA).

### 3.2. Substrate Preparation

Silicon wafers (Siegert Wafers, Germany) were used as reference substrates for developing and proving the success of the modifications utilizing various surface sensitive techniques. The developed modification avenues were translated to various substrates depending on the particular study, i.e., gold coated SPR chips (Institute of Photonics and Electronics, CAS, Czech Republic) to test the fouling resistance of the modifications, glass substrates for the cell studies, and woven PET grafts (Generous gift from VUP Medical, Czech Republic) as a proof-of-concept material. We have performed additional characterization of each modification step on every type of utilized substrate.

Before modification all substrates were rinsed with ethanol and water, blow dried with nitrogen, and activated in a UV-ozone cleaner for 20 min.

### 3.3. Immobilization of ATRP Initiator over the Reference Si Surfaces, Glass Substrates and SPR-Gold Coated Chips

The activated Si substrates were immersed in 0.1% *v*/*v* toluene solution of (11-(2-bromo-2-methyl)propionyloxy)undecyltrichlorosilane and kept at room temperature for 3 hours. After the immobilization reaction, the substrates bearing the ATRP initiator were thoroughly rinsed with toluene, acetone, ethanol, and water, and finally blow dried with nitrogen.

For the gold coated SPR chips, immediately after activation the samples were placed in a 1 mM solution of ω-mercaptoundecyl bromoisobutyrate in absolute ethanol and kept in the dark at room temperature overnight. Subsequently the samples were washed with ethanol and water and blow dried with nitrogen.

### 3.4. SI-ATRP of poly(MeOEGMA) Block

A flask containing CuBr_2_ (80.44 mg, 3.6 mmol), 2,2’-bipyridil (742.56 mg, 4.756 mmol) and CuBr (258.36 mg, 1.8 mmol) was degassed by flow of Ar for 1 hour before 24 ml of degassed MeOH was added and the mixture was stirred till full dissolution of the solids. Then degassed solution of oligo(ethylene glycol) methyl ether methacrylate (MeOEGMA, 27.3 g, 91.02 mmol) in 24 ml of water was added. The polymerization solution was transferred under Ar protection to the reactors containing the glass substrates, referent Si substrates and SPR chips with the self-assembled monolayers (SAM) of ATRP initiators. The reaction was allowed to proceed at 30 °C for 20 min. The polymerization was terminated by removing the chips from the polymerization solution. The substrates were rinsed twice with ethanol and water and blow dried with nitrogen.

### 3.5. SI-ATRP of poly(GMA) Block

A round-bottom flask containing *N*,*N*-dimethylformamide (DMF, 40 ml), glycidyl methacrylate (GMA, 26.8 ml, 196 mmol), 2,2’-bipyridyl (764 mg, 4.89 mmol) and CuBr_2_ (87.2 mg, 3.92 mmol) was degassed by flow of Ar for 1 hour before CuBr (280.4 mg, 1.96 mmol) was added under Ar atmosphere and the mixture was stirred till full dissolution of the solids. Then, the polymerization solution was transferred to the Ar filled reactors containing the glass substrates, SPR chips, and reference Si substrates coated with the bottom block of poly(MeOEGMA), and the reaction was allowed to proceed for 5 hours at 30 °C. The polymerization was terminated by removing the chips from the polymerization solution. The substrates were rinsed with DMF and dichloromethane and blow dried with nitrogen.

### 3.6. Functionalization of the Hierarchical Polymer Brushes with Azide Groups

Briefly, the 50 ml DMF solution of NaN_3_ (3.4 mg/ml) was injected to previously purged with Ar reactors containing the polymer-brush coated substrates. The reaction was allowed to proceed at 60°C for 24 h, then the reaction was terminated by removing of the silicon samples bearing the hierarchical poly(MeOEGMA-*block*-GMA-N_3_) brushes from the solution. The chips were rinsed with copious amounts of DMF and water and blow dried with nitrogen.

### 3.7. Peptide Synthesis, Radio-Labeling and Immobilization to poly(MeOEGMA-block-GMA-N_3_)

Pentynoyl–GGGRGDSGGGY–NH_2_ peptide was synthesized on TentaGel R Rink Amide resin (Rapp-Polymere, Germany) and its identity was confirmed by HPLC and MALDI–TOF MS analysis. The chloramine T/ascorbic acid radiolabeling method was adapted for the conditions of the solid–phase peptide ^125^Iodine radiolabeling according the reports of Proks et al. [38]. The immobilization of labeled pentynoic–GGGRGDSGGGY(^125^I)–NH_2_ and nonlabeled pentynoic–GGGRGDSGGGY–NH_2_ peptide to poly(MeOEGMA-*block*-GMA-N_3_) brushes was performed utilizing a copper-catalyzed Huisgen azide-alkyne ‘‘click’’ cycloaddition reaction from water solutions of molar concentrations ranging from of 0.3 to 1.5 × 10^3^ μM. The oxygen was removed from 1 mL peptide solution by purging with Ar. Subsequently 10 μL of 0.2% sodium ascorbate and 4 μL of 0.05 M CuSO_4_ were added to the solution. The poly(MeOEGMA-*block*-GMA-N_3_) brushes were initially swollen by putting the modified substrates over 100 μL water drops spread on parafilm for 15 min. Afterwards additional 100 μL of the peptide solution was added and the click reaction was left to proceed for 15 min (in order to limit the amount of non-covalently bound peptides). After the peptide immobilization, the substrates were washed with copious amounts of water. The nonspecific binding of peptides to poly(MeOEGMA-*block*-GMA-N_3_) brushes was accessed on samples exposed to the same binding conditions in the absence of copper catalyst. The surface concentration of immobilized peptides was determined directly by measuring sample activities using a Bqmetr 4 ionization chamber (Empos Ltd., Czech Republic) and a NaI/Tl SpectroAnalyzer, (AccuSync Medical Research Corporation, USA).

### 3.8. Adopting the Modification to the Surface of PET Woven Vascular Grafts

#### 3.8.1. Incorporation of Amine Groups via Aminolysis Reaction

The woven PET cylindrical samples (*ϕ* = 0.5 cm) were cut in 1-cm long pieces, sonicated in ethanol and water, and blow dried with nitrogen before immersing in the reaction solution. The reaction was performed with mixture of methanol and ethylenediamine at 50 °C with volume ratio MeOH:EtDA 60:40 and carried out for 30 min.

#### 3.8.2. Immobilization of ATRP Initiator

The initiator (α-bromoisobutyryl bromide, BiBB) was covalently attached to the modified grafts by acylation reaction of the present amino- and hydroxyl-groups. The samples were left in contact with 20 mL 0.24 M solution of triethylamine in dry THF at 0 °C and shaken for 10 min. Subsequently, 10 mL 0.24 M solution of BiBB in dry THF was added and the reaction was allowed to proceed for 3 min. before removing the samples from the solution. The solid residues were washed repeatedly with THF, ethanol, and water and the samples were blow dried with nitrogen.

#### 3.8.3. Polymerization and Functionalization with Peptides of the PET Vascular Grafts

All polymerization and modification steps were carried out under the same reaction conditions as on planar silicon substrates. The reagents concentrations and volume of reaction mixture used in the RGD immobilization reaction were recalculated using the data for the specific surface of the PET graft in order to provide identical conditions per unit area as in the case of the reference planar silicon substrates.

### 3.9. Spectroscopic Ellipsometry (SE)

The dry thickness of the obtained polymer layers after the polymerization steps was measured using a J.A. Woollam M-2000X Spectroscopic Ellipsometer (J.A. Woollam, USA). Data were acquired in the wavelength range *λ* = 245–1000 nm at angles of incidence of 60, 65, and 70°. The experimental data were fitted with multilayer models in the CompleteEASE software, using a Cauchy dispersion relation for the polymer layers.

### 3.10. Grazing Angle Attenuated Total Reflection Fourier-Transform Infrared Spectroscopy (GAATR-FTIR)

The IR spectra of all films were recorded in dry state utilizing a Nicolet Nexus 870 FTIR spectrometer (ThermoFisher Scientific, USA) equipped with VariGATR attachment (Harrick Scientific Products, USA). The attachment comprises Ge hemispherical ATR crystal and allows measurement at AOI in the range of 60-65°. The measurement chamber was continuously purged with dry air. The reported GAATR-FTIR spectra are averages of 256 scans taken with resolution of 2 cm^−1^. The acquisition time was about 3 min. All reported GAATR-FTIR spectra present the absorbance. The reference spectra were recorded on freshly cleaned silicon sample.

### 3.11. X-Ray Photoelectron Spectroscopy (XPS)

Measurements were performed using a K-Alpha^+^ XPS spectrometer (ThermoFisher Scientific, UK) operating at base pressure of 1.0 × 10^−7^ Pa. Data acquisition and processing were performed using the Thermo Avantage software. All samples were analyzed using a microfocused (spot size 400 μm), monochromated Al Kα X-ray radiation with pass energy of 200 eV for survey and 50 eV for high-energy resolution core level spectra. The X-ray angle of incidence was 30° and the emission angle was along the surface normal. The K-Alpha charge dual compensation system was employed during analysis, using electrons and low-energy argon ions to prevent localized charge build-up. The analyzer transmission function, Scofield sensitivity factors, and effective attenuation lengths (EALs) for photoelectrons were applied for quantification. EALs were calculated using the standard TPP-2 M formalism. The binding energy scale of the XPS spectrometer was calibrated by the well-known positions of the C 1s C–C and C–H, C–O and C(=O)–O peaks of polyethylene terephthalate and Cu 2p, Ag 3d, and Au 4f peaks of Cu, Ag and Au metals, respectively. All measured spectra were charge referenced to the C 1s contribution at binding energy of 285.0 eV attributed to C–C and C–H moieties. The obtained high-resolution spectra were fitted with Voigt profiles to probe the individual contributions of present chemical species.

### 3.12. Surface Plasmon Resonance Spectroscopy (SPR)

SPR fouling measurements were carried out using an instrument (Institute of Photonics and Electronics, CAS, Czech Republic) based on the Kretschmann configuration and spectral interrogation. In all the experiments the temperature was kept at 25 °C and the flow rate of the solutions was 20 μL/min. The amount of protein mass immobilized on the surface is calculated from the difference in the resonance wavelength before and after contact with the test solutions. Under the conditions used, an increase of 1 nm in the resonance wavelength *λ*_res_ is attributed to a mass increase of 15 ng/cm^2^ on the measured surface. The SPR chips, already coated with polymer and RGD functionalized polymer brushes, were placed in the SPR setup. Resistance to fouling was measured by first establishing a stable baseline by flowing PBS on the surface until a stable baseline was obtained. It was then replaced, then replacing it with undiluted human blood plasma for 10 min and subsequently PBS was flowed again. The procedure was repeated with the cell growth media used in the cell experiments.

### 3.13. Scanning Electron Microscopy (SEM)

The SEM analysis was performed on a Quanta 200 FEG (FEI, Czech Republic) microscope. All presented micrographs are secondary electron images taken under high vacuum using an accelerating voltage of 30 kV. The magnification of the presented micrographs is ×2500.

### 3.14. Analysis of Specific Surface Area

The specific surface area was measured by nitrogen adsorption technique on a Gemini VII 2390 (Micromeritics Instruments Corp., USA) device. Before the analysis, the PET woven graft was vacuum degassed at RT for 16 h. The surface area was calculated from the Brunauer–Emmett–Teller (BET) adsorption/desorption isotherm using Gemini software.

Accordingly determined porosity and specific surface area of the vascular graft was determined to be 66% and the 0.76 m^2^/g, respectively. The determined parameters were used to provide the same reaction conditions per unit area as in the case of the planar surfaces.

### 3.15. Cell Seeding Experiments

The system developed in this work allows us to study in detail the effect that the nature of the surface has on the interaction between tissues (cells) and artificial surface. For this purpose, we performed a series of cell-based experiments over the protein-repulsive polymer layers and the peptide-modified adhesive layers. Two different types of experiments were performed. First, a live/dead assay was used to distinguish the number of live cells from number of dead cells on the given surface. The assay discriminates live cells with an intact plasma membrane by staining intracellular esterase activity from dead cells which bind ethidium homodimer-1 to exposed DNA. Secondly, morphological experiments were focused on affinity of the cells to the surface. Human umbilical vein endothelial cells (HUVECs) were used for testing of five different types of surface under static conditions in both experiments.

Cell culture plate (tissue culture treated polystyrene), PET foil and bare cover glass were used as control surfaces. Glass samples modified with two different surface concentration of RGD fragment (3.2 × 10^2^ nmol/cm^2^ and 1.2 × 10^3^ nmol/cm^2^) were selected as cell-adhesive surface, and non-modified diblock poly(MeOEGMA-*block*-GMA-N_3_) as antifouling polymer support. All tested surfaces were sterilized by 70% ethanol for 20 min and washed by PBS. Subsequently, samples were transferred into the 12-well plate (TPP, Switzerland). HUVEC were seeded in concentration 5.000 cells per cm^2^. The Live/Dead assay was performed after 3 days of the cultivation at 37 °C, 5% CO_2_ atmosphere using Endothelial Cell Growth Medium 2 Kit. Mixture of 1.5 µM Calcein and 2 µM ethidium homodimer-1 (final concentration) was directly added to cell culture media with no additional washing steps. Cell viability was tested in three independent measurements/biological replicates. Every measurement included five replicates of samples having the same surface modification. Four images were made from each sample. Number of live cells was manually calculated from images in ImageJ using the Cell Counter plugin. Total cell number (*T_n_*) was determined as $T_{n}=cell\;number\times \frac{glass\;area}{image\;area}$. Significant differences between the number of cells adhered on the tested surfaces were accessed by the student t-test with probability value *p* < 0.05. For morphology observation, HUVEC were fixed for staining using cold methanol (T = −20 °C) for 10 min. Afterwards, adhered cells were stained by Phalloidin Atto 594 (F-actin) and Hoechst 33342 (nuclei). Cell morphology was observed under confocal microscope Olympus IX83 (Japan).

The same conditions and methods (surface sterilization treatments, cell concentration, cultivation media and conditions, cell fixation and microscopic observations) were utilized for monitoring of the cell adhesion and morphology of HUVECs on woven PET conduits modified with anti-fouling diblock copolymer and its bioactive counterpart with 1.2 × 10^3^ nmol/cm^2^ RGD peptides. The tested grafts were immobilized to the bottom of the 24-well plates by Scaffdex-CellCrown 24 (Scaffdex Oy, Finland) before cell seeding.

## 4. Conclusions

In this contribution, we present the surface modification of woven PET vascular grafts via the direct synthesis of hierarchical antifouling brushes bearing ECM-mimetic peptides to address the main problems for endothelialization. Namely, the coating suppressed the non-specific protein adsorption from blood and cell-seeding medium, offering a potential avenue for the suppression of deleterious responses, and at the same time provided excellent cell adhesion properties. The design of the brush and selection of the CuAAC for peptide immobilization ensured the preservation of the non-fouling properties after peptide immobilization. The chosen type of modification reaction is highly selective, easy to tune, and gives no side products which could complicate the purification process. Control over the immobilization of the peptide sequences in a wide range of surface concentrations was achieved, which in turn enabled the stable adhesion and growth of the cell cultures chosen as model for testing in situ endothelialization over the surface. The HUVEC cells formed a continuous and stable layer oriented along the PET fibers modified with the antifouling hierarchical poly(MeOEGMA-*block*-GMA-N_3_) polymer brushes bearing 1.2 × 10^3^ nmol/cm^2^ RGD peptides. The cells colonized the woven conduit and interpenetrated the biofunctional mesh. In perspective, varying of the biomimetic peptide sequence can be utilized in the attachment of different cell types, offering a flexible and straightforward synthetic route for the modification of polymer layers and a promising tool for designing new in situ tissue regeneration constructs.

## Supplementary Materials

Supplementary Materials can be found at https://www.mdpi.com/1422-0067/21/18/6800/s1. The supporting information reports the structure of the RGD sequence, the synthetic route to incorporation of SI-ATRP initiator over the PET graft, surface composition on reference silicon and PET vascular graft surfaces after individual steps of polymerization, modification and RGD immobilization, high-resolution Br 3d XPS spectra of reference silicon and aminolyzed PET graft bearing initiator moieties, SEM analysis, GAATR-FTIR spectra of PET vascular graft after individual modification steps, supporting confocal micrograph.
