# Supplementary material for: Surface Design of Antifouling Vascular Constructs Bearing Biofunctional Peptides for Tissue Regeneration Applications
Source: Int J Mol Sci. 2020 Sep 16;21(18):6800. doi: 10.3390/ijms21186800 (PMC7554689; doi:10.3390/ijms21186800)
Supplement: Supplementary file 1 [file ijms-21-06800-s001.pdf]

Supporting information for

Surface design of antifouling vascular constructs bearing biofunctional peptides for tissue regeneration applications

Radoslava Sivkova, Johanka Táborská, Alain Reparaz, Andres de los Santos Pereira, Ilya Kotelnikov, Vladimir Proks, Jan Kučka, Tomáš Riedel, Jan Svoboda and Ognen Pop-Georgievski

Scheme S1. Structure of the RGD sequence

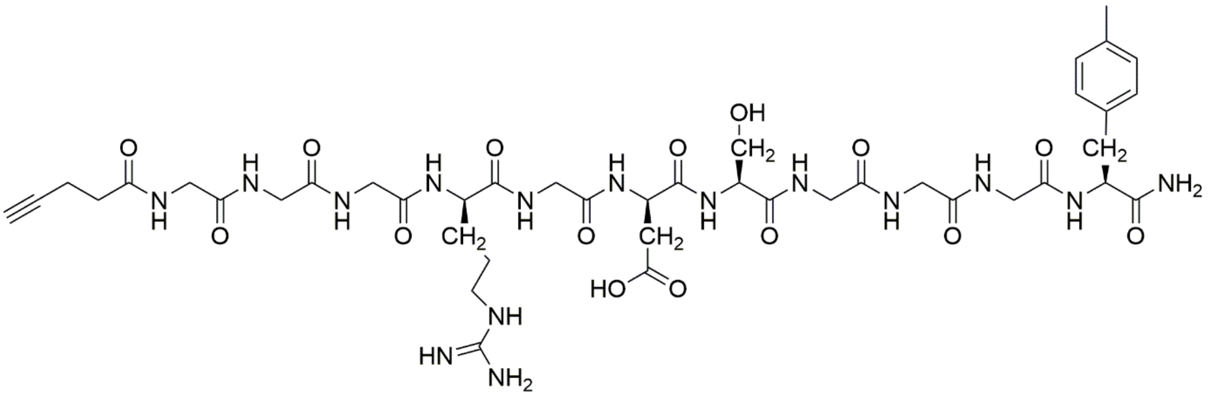

Scheme S2. Synthetic route to incorporation of SI-ATRP initiator over the PET graft

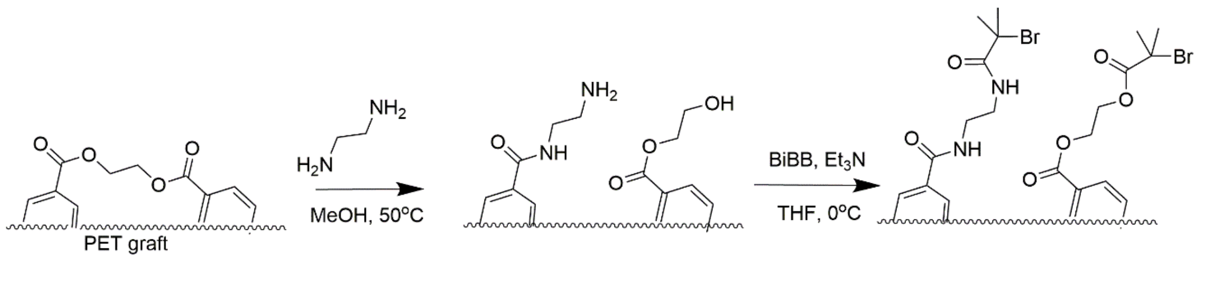

Table S1. Atomic % of chemical moieties present on the planar silicon surfaces as determined by XPS.

	C 1s C-C	C 1s C-O	C 1s N-C=O	C 1s C(=O)-O	N 1s azide N^-	N 1s azide N^+	N 1s peptide	N 1s triazole, guanidinine	O 1s	Br 3d	Si 2p
RGD 4.6×10^3 nmol/cm^2	15.8	40.2	3.7	4.5			7.2	0.5	28.0		
RGD 1.2×10^3 nmol/cm^2	15.4	43.8	2.1	5.0			3.7	0.3	29.8		
RGD 3.2×10^2 nmol/cm^2	15.5	45.9	0.2	5.3	1.5	0.8	0.4	0.1	30.3		
RGD 12.1 nmol/cm^2	15.6	46.0		5.3	1.8	0.9			30.3		
RGD 1.1 nmol/cm^2	15.4	46.5		5.5	1.3	0.7			30.7		
poly(MeOEGMA-*block*-GMA-N_3)	14.7	46.6		5.3	2.1	1.1			30.1		
poly(MeOEGMA-*block*-GMA)	15.0	46.6		6.7					31.7		
poly(MeOEGMA)	13.3	49.7		5.0					31.6	0.4	
Initiator	21.3	5.3		1.6					31.2	1.2	39.5

* Cu 2p was under detection limit (<0.1%

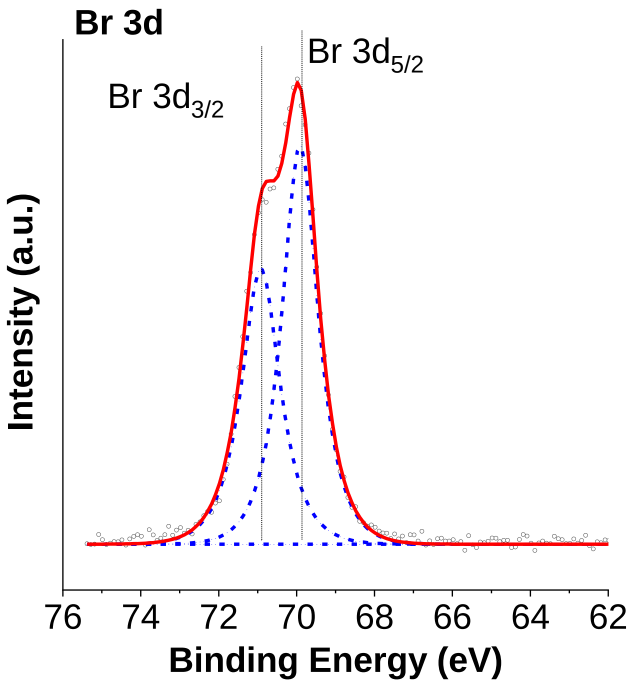

Figure S1. High resolution Br 3d XPS spectrum of ATRP initiator immobilized on silicon substrate

Characterization of poly(MeOEGMA-*block*-GMA-N_3) hierarchical brushes grown from activated woven PET vascular grafts

The individual modification steps for the synthesis of biofunctional hierarchical poly(MeOEGMA-*co*-GMA-N_3) polymer brushes bearing 1.2×10^3 nmol/cm^2 RGD were followed via XPS, GAATR-FTIR and SEM analysis (**Figure S2-S5** and **Table S2**).

The incorporated reactive groups were acylated with α-bromoisobutyryl bromide (BiBB) to provide SI-ATRP initiating moieties over the graft surface. The SI-ATRP polymerization and modification conditions optimized on planar surfaces were applied on the activated PET grafts.

The high-resolution C 1s XPS spectrum of PET grafts (**Figure S2** and **Table S2**) showed three peaks assigned to C–C (285.0 eV), C–O (286.6 eV) and O–C=O (288.9 eV) functionalities. Anchoring amine and hydroxyl groups on the woven vascular grafts were inserted through an aminolysis reaction. The conditions of this reaction were optimized to minimize any deterioration of the structure of the woven PET fibers, which was observed via SEM (Figure S4). In the XPS analysis, the aminolysis reaction of the PET woven material led to the appearance of a nitrogen peak at 400 eV originating from the incorporated amide and amine moieties. The changes induced to the C 1s spectrum were mainly seen through the increase of the peak width, since amide contributions were superimposed with the O–C=O moiety and C–N contributions overlap with the C–O component. Importantly, the acylation of the available amine and hydroxyl groups gave the characteristic Br $3d_{5/2}$—Br $3d_{3/2}$ spin split doublet at about 70 eV (separation between components of 1 eV, **Figure S3**).

Further analysis was performed via GAATR-FTIR (**Figure S5**). The FTIR spectrum of the initiator-functionalized PET mesh is strongly dominated by the contributions of the bulk material. The synthesis of hierarchical poly(MeOEGMA-*block*-GMA-N_3) brushes led to broadening of the main peaks of the PET woven mesh. We could identify the main peaks of MeOEGMA at about 1160 cm^{-1} as shoulders on the dominating peaks of the mesh. Notably a diagnostic azide stretching band at 2103 cm^{-1} appeared in the spectra of the hierarchical brush, proving the successful ring opening of the GMA blocks.

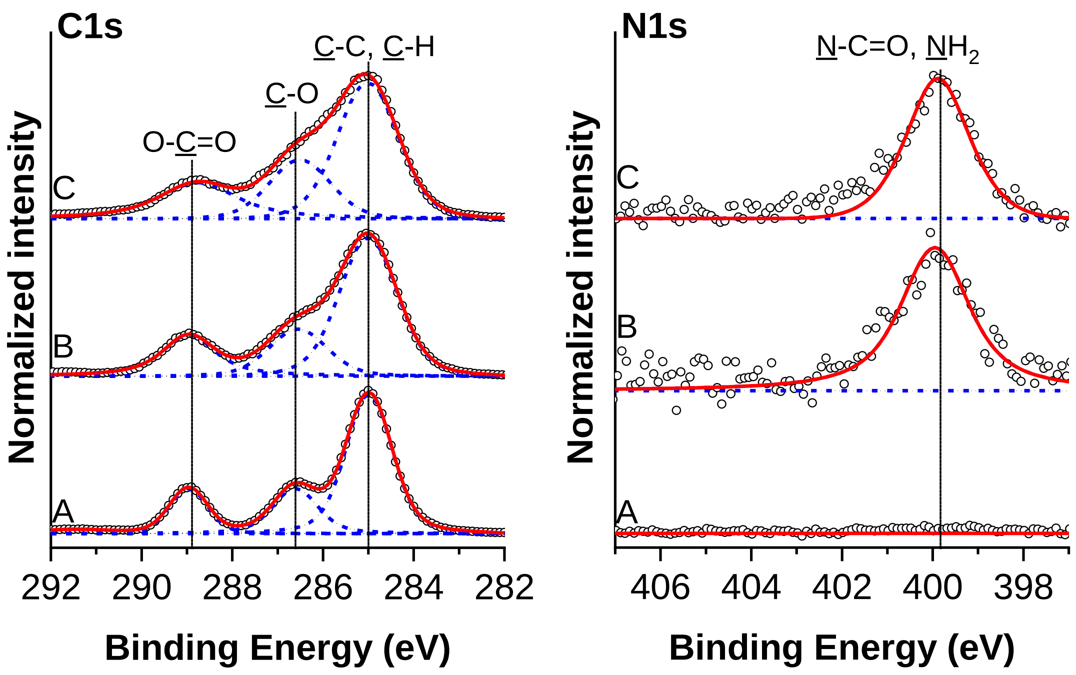

Figure S2. High resolution C 1s and N 1s XPS spectra of pristine vascular PET graft (A), graft after aminolysis (B), and aminolyzed graft bearing bromoisobutyrate ATRP initiating moieties (C), graft bearing the hierarchical poly(MeOEGMA-*block*-GMA-N_3) brushes. The composition of the layers is presented in **SI**, **Table S2**.

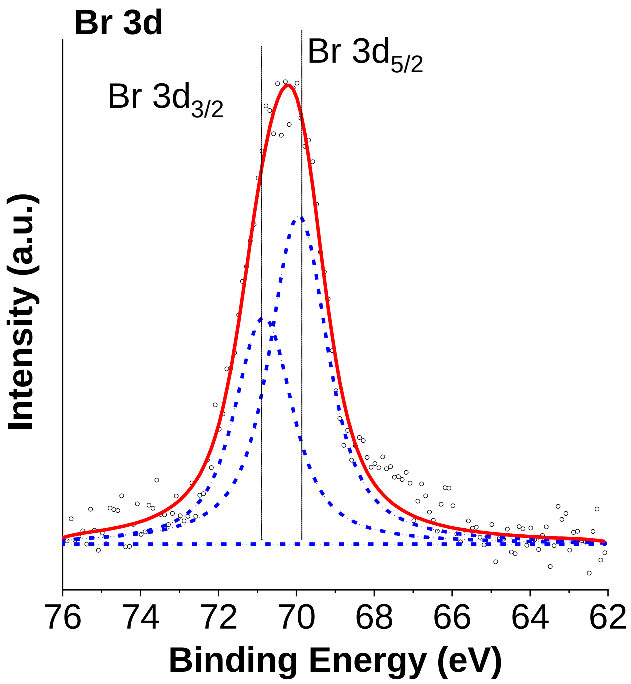

Figure S3. High resolution Br 3d XPS spectra graft after immobilization of initiator on the aminolyzed vascular PET graft.

Table S2. Atomic % of chemical moieties present on the surface as determined by XPS.

	C 1s C-C	C 1s C-O	C 1s C(=O)-N	C 1s C(=O)-O	N 1s N-C(=O), NH_2	N 1s N^-	N 1s N^+	N 1s triazole, guanidinine	Br 3d	O 1s
RGD 1.2×10^3 nmol/cm^2	21.4	30.5	4.2	9.0	6.1			1.0		27.8
poly(MeOEGMA-*block*-GMA-N_3)	23.5	30.4		12.7		3.1	1.5			28.8
Aminolyzed PET graft with BiBB	35.3	15.3		15.0	2.0				0.8	31.6
Aminolyzed PET graft	44.0	14.1		11.0	1.8					29.1
PET graft	44.9	15.1		11.3						28.7

* Cu 2p was under detection limit (<0.1%)

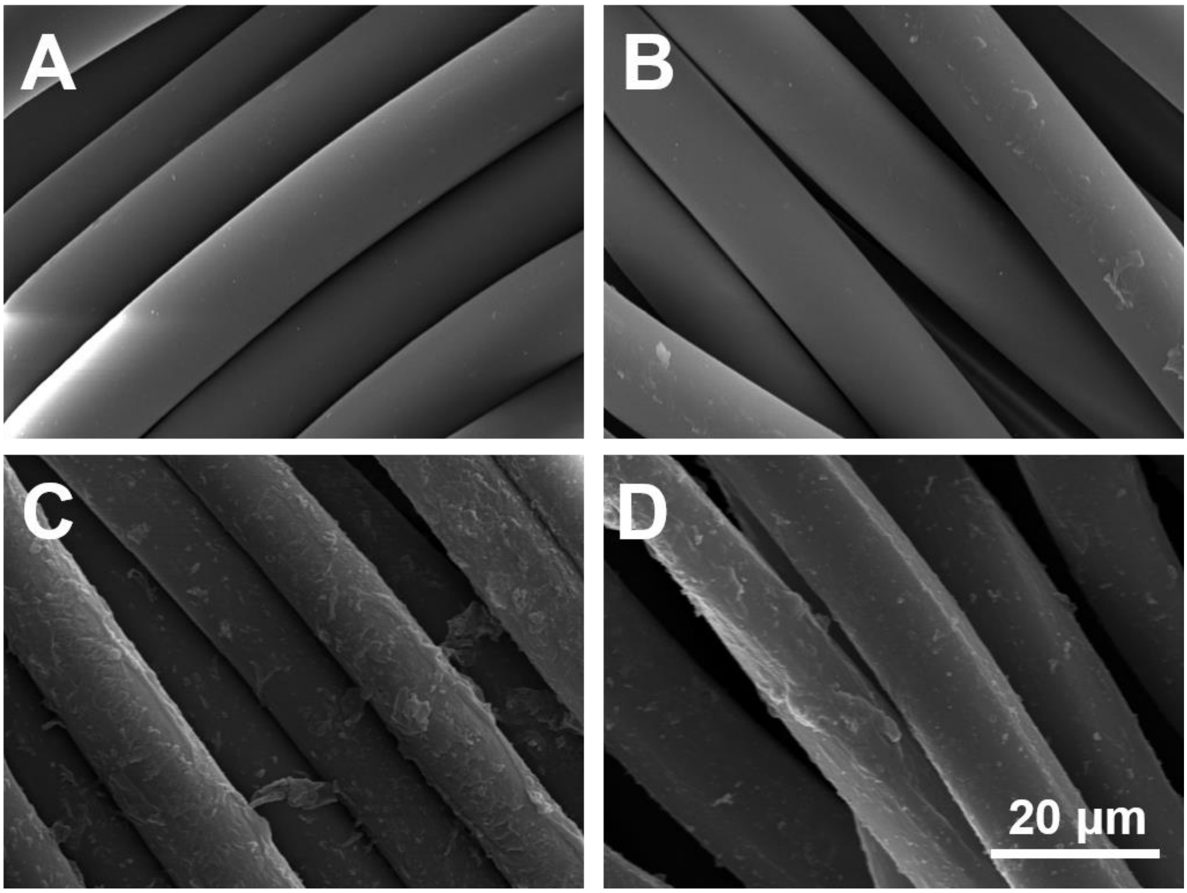

Figure S4. Scanning electron micrographs of pristine PET vascular graft (A) and grafts after aminolysis (B), SI-ATRP synthesis of hierarchical poly(MeOEGMA-*block*-GMA-N_3) polymer brush (C) and subsequent „click“ of 1.2×10^3 nmol/cm^2 RGD peptides. All treated samples show rather uniform topography without disruptions of the fiber continuous structure. All surface modifications were restricted to the most outer surface of the individual fibers, thus preserving the initial integrity and mechanical stability of the woven PET material.

.

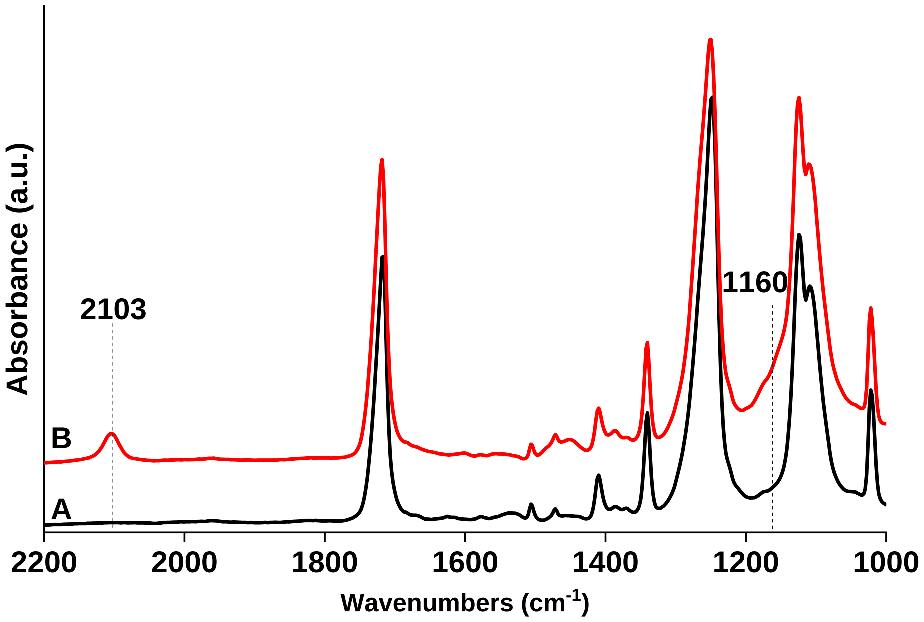

Figure S5. GAATR-FTIR spectra of PET vascular graft after aminolysis and reaction with initiator (A) and after the synthesis of anti-fouling hierarchical poly(MeOEGMA-*block*-GMA-N_3) polymer brushes (B).

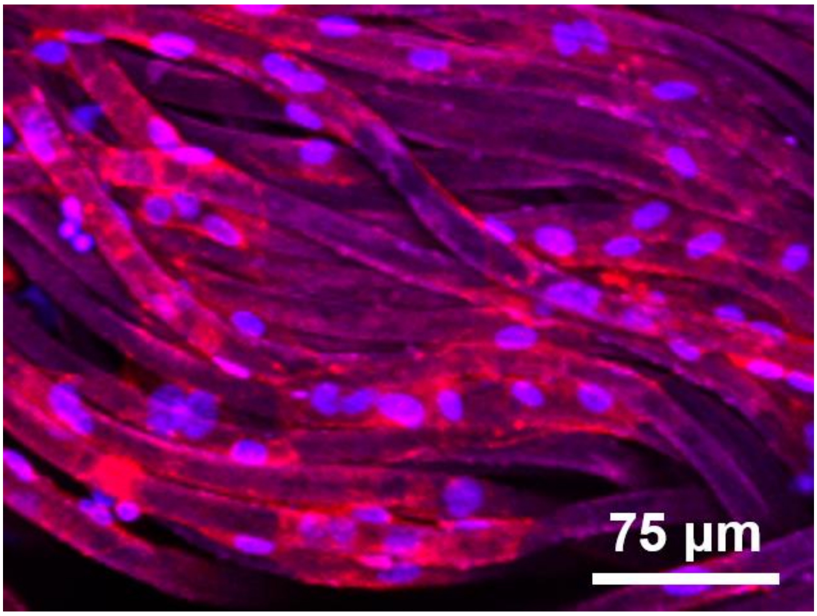

Figure S6. Adhesion of HUVEC cells on the outer side of the biofunctional hierarchical poly(MeOEGMA-*block*-GMA-N_3) polymer brushes bearing 1.2 × 10^3 nmol/cm^2 RGD after 5 days of seeding. The actin filaments (red) and cell nuclei (blue) were stained by Phalloidin–Atto 594 and Hoechst 33342, respectively.
